# 4-(4-Meth­oxy­phen­yl)-2-oxo-1,2,5,6-tetra­hydro­benzo[*h*]quinoline-3-carbo­nitrile

**DOI:** 10.1107/S1600536811033897

**Published:** 2011-08-27

**Authors:** Abdullah M. Asiri, Hassan M. Faidallah, Abdulrahman O. Al-Youbi, Khalid A. Alamry, Seik Weng Ng

**Affiliations:** a Chemistry Department, Faculty of Science, King Abdulaziz University, PO Box 80203 Jeddah, Saudi Arabia; b Department of Chemistry, University of Malaya, 50603 Kuala Lumpur, Malaysia

## Abstract

In the mol­ecule of the title compound, C_21_H_16_N_2_O_2_, the tetra­hydro­benzo[*h*]quinoline fused-ring system is buckled owing to the ethyl­ene –CH_2_CH_2_– fragment, the benzene ring and the pyridine ring being twisted by 19.7 (1)°. The 4-substituted aromatic ring is bent away from the pyridine ring by 50.3 (1)° in order to avoid crowding the cyanide substituent. In the crystal, two mol­ecules are linked by a pair of N—H⋯O hydrogen bonds to form a centrosymmetric dimer.

## Related literature

For background to the anti­cancer properties of this class of compounds, see: Rostom *et al.* (2011 ▶).
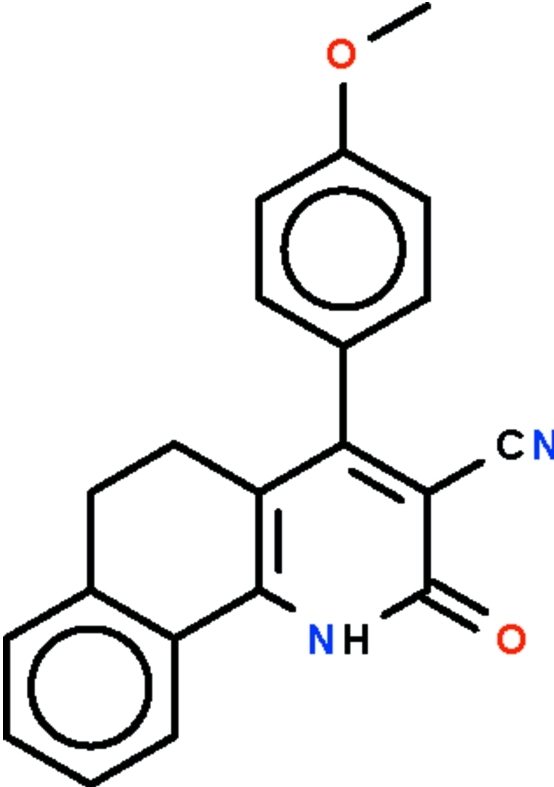


## Experimental

### 

#### Crystal data

C_21_H_16_N_2_O_2_
*M* *_r_* = 328.36
Monoclinic, 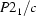

*a* = 14.2016 (2) Å
*b* = 14.4725 (2) Å
*c* = 7.9935 (1) Å
β = 96.017 (1)°
*V* = 1633.87 (4) Å^3^
*Z* = 4
Cu *K*α radiation
μ = 0.70 mm^−1^
*T* = 100 K
0.30 × 0.25 × 0.20 mm

#### Data collection

Agilent SuperNova Dual diffractometer with Atlas detector
Absorption correction: multi-scan (*CrysAlis PRO*; Agilent, 2010 ▶) *T*_min_ = 0.818, *T*_max_ = 0.873
6187 measured reflections
3211 independent reflections
3011 reflections with *I* > 2σ(*I*)
*R*_int_ = 0.014

#### Refinement

*R*[*F* ^2^ > 2σ(*F* ^2^)] = 0.035
*wR*(*F* ^2^) = 0.096
*S* = 1.03
3211 reflections
230 parameters
H atoms treated by a mixture of independent and constrained refinement
Δρ_max_ = 0.21 e Å^−3^
Δρ_min_ = −0.21 e Å^−3^

### 

Data collection: *CrysAlis PRO* (Agilent, 2010 ▶); cell refinement: *CrysAlis PRO*; data reduction: *CrysAlis PRO*; program(s) used to solve structure: *SHELXS97* (Sheldrick, 2008 ▶); program(s) used to refine structure: *SHELXL97* (Sheldrick, 2008 ▶); molecular graphics: *X-SEED* (Barbour, 2001 ▶); software used to prepare material for publication: *publCIF* (Westrip, 2010 ▶).

## Supplementary Material

Crystal structure: contains datablock(s) global, I. DOI: 10.1107/S1600536811033897/xu5292sup1.cif

Structure factors: contains datablock(s) I. DOI: 10.1107/S1600536811033897/xu5292Isup2.hkl

Supplementary material file. DOI: 10.1107/S1600536811033897/xu5292Isup3.cml

Additional supplementary materials:  crystallographic information; 3D view; checkCIF report

## Figures and Tables

**Table 1:** Hydrogen-bond geometry (Å, °)

*D*—H⋯*A*	*D*—H	H⋯*A*	*D*⋯*A*	*D*—H⋯*A*
N1—H1⋯O1^i^	0.90 (2)	1.94 (2)	2.823 (1)	166 (1)

Symmetry code: (i) 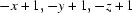.

**Fig. 1.**
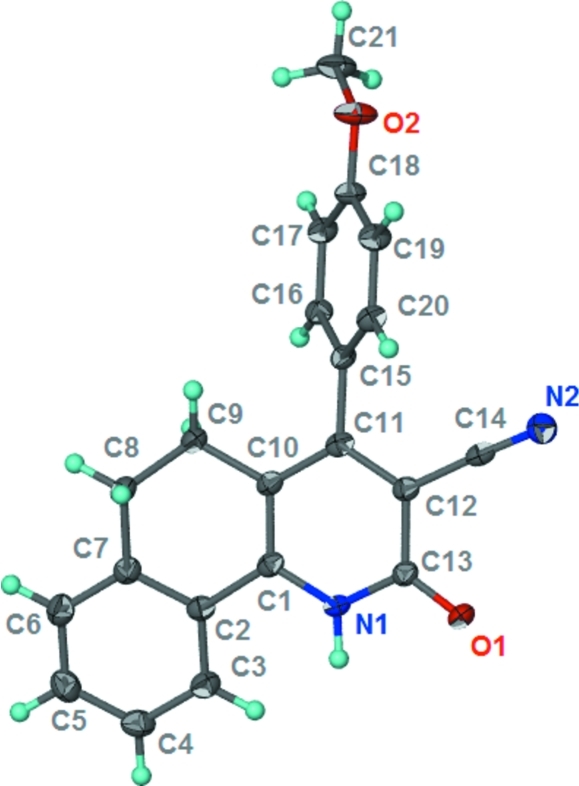
Thermal ellipsoid plot (Barbour, 2001) of C21H16N2O2 at the 70% probability level; hydrogen atoms are drawn as spheres of arbitrary radius.

## supplementary crystallographic information

### Comment

The compound (Scheme I) belongs to a series of cyano-pyridinones that have been evaluated for their anticancer properties (Rostom *et al.*, 2011). The tetrahydrobenzo[*h*]quinoline fused-ring system is buckled owing to the ethylene –CH_2_CH_2_– fragment, the benzene ring and the pyridine ring being twisted by 19.7 (1)°. The 4-subsituted aromatic ring is bent away from the pyridine ring by 50.3 (1) ° in order to avoid crowding the cyanide substituent (Fig. 1). Two molecules are linked by an N—H···O hydrogen bonds to form a centrosymmetric dimer (Table 1).

### Experimental

A mixture of *p*-anisaldehyde (1.36 g, 10 mmol), 1-tetralone (1.46 g, 10 mmol), ethyl cyanoacetate (1.1 g, 10 mmol) and ammonium acetate (6.2 g, 80 mmol) in absolute ethanol (50 ml) was refluxed for 6 h. The reaction mixture was allowed to cool, and the yellow precipitate that formed was filtered, washed with water, dried and recrystallized from ethanol; m.p. 587–589 K.

### Refinement

Carbon- and nitrogen-bound H atoms were placed in calculated positions [C—H 0.95 to 0.99 Å, *U*_iso_(H) = 1.2*U*_eq_(C)] and were included in the refinement in the riding model approximation.

The amino H atom was located in a difference Fourier map and was freely refined.

### Crystal data

**Table:**

C_21_H_16_N_2_O_2_	*F*(000) = 688
*M**_r_* = 328.36	*D*_x_ = 1.335 Mg m^−^^3^
Monoclinic, *P*2_1_/*c*	Cu *K*α radiation, λ = 1.54184 Å
Hall symbol: -P 2ybc	Cell parameters from 4172 reflections
*a* = 14.2016 (2) Å	θ = 3.1–74.1°
*b* = 14.4725 (2) Å	µ = 0.70 mm^−^^1^
*c* = 7.9935 (1) Å	*T* = 100 K
β = 96.017 (1)°	Prism, yellow
*V* = 1633.87 (4) Å^3^	0.30 × 0.25 × 0.20 mm
*Z* = 4	

### Data collection

**Table:**

Agilent SuperNova Dual diffractometer with Atlas detector	3211 independent reflections
Radiation source: SuperNova (Cu) X-ray Source	3011 reflections with *I* > 2σ(*I*)
mirror	*R*_int_ = 0.014
Detector resolution: 10.4041 pixels mm^-1^	θ_max_ = 74.2°, θ_min_ = 4.4°
ω scans	*h* = −17→14
Absorption correction: multi-scan (*CrysAlis PRO*; Agilent, 2010)	*k* = −9→17
*T*_min_ = 0.818, *T*_max_ = 0.873	*l* = −9→9
6187 measured reflections	

### Refinement

**Table:**

Refinement on *F*^2^	Primary atom site location: structure-invariant direct methods
Least-squares matrix: full	Secondary atom site location: difference Fourier map
*R*[*F*^2^ > 2σ(*F*^2^)] = 0.035	Hydrogen site location: inferred from neighbouring sites
*wR*(*F*^2^) = 0.096	H atoms treated by a mixture of independent and constrained refinement
*S* = 1.03	*w* = 1/[σ^2^(*F*_o_^2^) + (0.0566*P*)^2^ + 0.481*P*] where *P* = (*F*_o_^2^ + 2*F*_c_^2^)/3
3211 reflections	(Δ/σ)_max_ = 0.001
230 parameters	Δρ_max_ = 0.21 e Å^−^^3^
0 restraints	Δρ_min_ = −0.21 e Å^−^^3^

### Fractional atomic coordinates and isotropic or equivalent isotropic displacement parameters (Å^2^)

**Table:**

	*x*	*y*	*z*	*U*_iso_*/*U*_eq_	
O1	0.38937 (5)	0.48227 (6)	0.53692 (9)	0.01885 (19)	
O2	−0.13689 (6)	0.71903 (6)	0.05350 (12)	0.0263 (2)	
N1	0.42821 (6)	0.55604 (6)	0.30260 (11)	0.0154 (2)	
H1	0.4886 (11)	0.5401 (11)	0.3377 (19)	0.026 (4)*	
N2	0.15375 (7)	0.53084 (7)	0.58833 (12)	0.0215 (2)	
C1	0.40584 (8)	0.60068 (7)	0.15327 (13)	0.0150 (2)	
C2	0.48272 (8)	0.62138 (7)	0.04885 (13)	0.0159 (2)	
C3	0.57808 (8)	0.62155 (8)	0.11363 (14)	0.0186 (2)	
H3	0.5951	0.6082	0.2292	0.022*	
C4	0.64792 (8)	0.64118 (8)	0.00983 (15)	0.0218 (2)	
H4	0.7127	0.6404	0.0540	0.026*	
C5	0.62308 (9)	0.66197 (8)	−0.15877 (15)	0.0228 (3)	
H5	0.6708	0.6763	−0.2294	0.027*	
C6	0.52862 (8)	0.66177 (8)	−0.22408 (14)	0.0208 (2)	
H6	0.5122	0.6759	−0.3395	0.025*	
C7	0.45770 (8)	0.64121 (7)	−0.12269 (13)	0.0168 (2)	
C8	0.35509 (8)	0.63521 (8)	−0.19115 (13)	0.0184 (2)	
H8A	0.3387	0.5700	−0.2181	0.022*	
H8B	0.3447	0.6714	−0.2965	0.022*	
C9	0.29074 (8)	0.67172 (8)	−0.06574 (13)	0.0178 (2)	
H9A	0.2994	0.7393	−0.0531	0.021*	
H9B	0.2239	0.6599	−0.1085	0.021*	
C10	0.31283 (7)	0.62548 (7)	0.10368 (13)	0.0151 (2)	
C11	0.24237 (7)	0.60637 (7)	0.21242 (13)	0.0150 (2)	
C12	0.26806 (7)	0.55905 (7)	0.36226 (13)	0.0149 (2)	
C13	0.36372 (7)	0.52863 (7)	0.40914 (13)	0.0151 (2)	
C14	0.20310 (7)	0.54250 (7)	0.48475 (13)	0.0159 (2)	
C15	0.14293 (7)	0.63712 (7)	0.16970 (13)	0.0153 (2)	
C16	0.12282 (8)	0.72862 (7)	0.12434 (13)	0.0161 (2)	
H16	0.1736	0.7709	0.1196	0.019*	
C17	0.03069 (8)	0.75948 (8)	0.08598 (14)	0.0177 (2)	
H17	0.0184	0.8223	0.0569	0.021*	
C18	−0.04357 (7)	0.69700 (8)	0.09073 (14)	0.0181 (2)	
C19	−0.02511 (8)	0.60501 (8)	0.13544 (14)	0.0193 (2)	
H19	−0.0759	0.5626	0.1386	0.023*	
C20	0.06719 (8)	0.57570 (8)	0.17509 (13)	0.0174 (2)	
H20	0.0793	0.5132	0.2063	0.021*	
C21	−0.15928 (8)	0.81389 (9)	0.01842 (17)	0.0261 (3)	
H21A	−0.2279	0.8207	−0.0075	0.039*	
H21B	−0.1275	0.8344	−0.0781	0.039*	
H21C	−0.1378	0.8516	0.1169	0.039*	

### Atomic displacement parameters (Å^2^)

**Table:**

	*U*^11^	*U*^22^	*U*^33^	*U*^12^	*U*^13^	*U*^23^
O1	0.0173 (4)	0.0238 (4)	0.0149 (4)	0.0045 (3)	−0.0007 (3)	0.0043 (3)
O2	0.0117 (4)	0.0218 (4)	0.0445 (5)	0.0017 (3)	−0.0010 (3)	0.0067 (4)
N1	0.0124 (4)	0.0186 (5)	0.0147 (4)	0.0027 (3)	−0.0015 (3)	0.0015 (3)
N2	0.0212 (5)	0.0219 (5)	0.0217 (5)	0.0001 (4)	0.0034 (4)	0.0016 (4)
C1	0.0168 (5)	0.0133 (5)	0.0141 (5)	0.0009 (4)	−0.0014 (4)	−0.0011 (4)
C2	0.0160 (5)	0.0136 (5)	0.0178 (5)	0.0014 (4)	0.0004 (4)	0.0001 (4)
C3	0.0179 (5)	0.0175 (5)	0.0199 (5)	0.0004 (4)	−0.0005 (4)	0.0024 (4)
C4	0.0157 (5)	0.0212 (6)	0.0281 (6)	−0.0009 (4)	0.0009 (4)	0.0022 (5)
C5	0.0221 (6)	0.0220 (6)	0.0254 (6)	−0.0017 (4)	0.0079 (4)	0.0020 (4)
C6	0.0252 (6)	0.0194 (5)	0.0181 (5)	0.0008 (4)	0.0032 (4)	0.0019 (4)
C7	0.0191 (5)	0.0143 (5)	0.0167 (5)	0.0017 (4)	0.0009 (4)	−0.0002 (4)
C8	0.0189 (5)	0.0224 (5)	0.0134 (5)	0.0023 (4)	−0.0009 (4)	0.0013 (4)
C9	0.0173 (5)	0.0208 (5)	0.0148 (5)	0.0033 (4)	−0.0004 (4)	0.0019 (4)
C10	0.0155 (5)	0.0151 (5)	0.0142 (5)	0.0011 (4)	−0.0013 (4)	−0.0006 (4)
C11	0.0157 (5)	0.0128 (5)	0.0156 (5)	0.0001 (4)	−0.0023 (4)	−0.0025 (4)
C12	0.0144 (5)	0.0147 (5)	0.0152 (5)	0.0005 (4)	−0.0003 (4)	−0.0010 (4)
C13	0.0162 (5)	0.0151 (5)	0.0138 (5)	0.0015 (4)	−0.0002 (4)	−0.0010 (4)
C14	0.0153 (5)	0.0141 (5)	0.0172 (5)	0.0014 (4)	−0.0038 (4)	−0.0003 (4)
C15	0.0146 (5)	0.0180 (5)	0.0128 (5)	0.0012 (4)	−0.0012 (4)	−0.0007 (4)
C16	0.0139 (5)	0.0175 (5)	0.0165 (5)	−0.0018 (4)	−0.0006 (4)	−0.0004 (4)
C17	0.0172 (5)	0.0155 (5)	0.0199 (5)	0.0009 (4)	−0.0003 (4)	0.0015 (4)
C18	0.0119 (5)	0.0206 (6)	0.0212 (5)	0.0016 (4)	−0.0013 (4)	0.0011 (4)
C19	0.0147 (5)	0.0186 (5)	0.0240 (6)	−0.0030 (4)	−0.0003 (4)	0.0006 (4)
C20	0.0179 (5)	0.0153 (5)	0.0183 (5)	0.0004 (4)	−0.0011 (4)	0.0008 (4)
C21	0.0168 (5)	0.0229 (6)	0.0383 (7)	0.0060 (5)	0.0012 (5)	0.0064 (5)

### Geometric parameters (Å, °)

**Table:**

O1—C13	1.2443 (13)	C8—H8B	0.9900
O2—C18	1.3653 (13)	C9—C10	1.5137 (14)
O2—C21	1.4304 (14)	C9—H9A	0.9900
N1—C1	1.3652 (13)	C9—H9B	0.9900
N1—C13	1.3729 (14)	C10—C11	1.4197 (15)
N1—H1	0.904 (16)	C11—C12	1.3947 (15)
N2—C14	1.1519 (15)	C11—C15	1.4859 (14)
C1—C10	1.3859 (15)	C12—C14	1.4341 (15)
C1—C2	1.4729 (15)	C12—C13	1.4399 (14)
C2—C3	1.3984 (15)	C15—C16	1.3947 (15)
C2—C7	1.4095 (15)	C15—C20	1.3994 (15)
C3—C4	1.3875 (16)	C16—C17	1.3860 (15)
C3—H3	0.9500	C16—H16	0.9500
C4—C5	1.3898 (17)	C17—C18	1.3928 (16)
C4—H4	0.9500	C17—H17	0.9500
C5—C6	1.3875 (17)	C18—C19	1.3961 (16)
C5—H5	0.9500	C19—C20	1.3826 (15)
C6—C7	1.3896 (16)	C19—H19	0.9500
C6—H6	0.9500	C20—H20	0.9500
C7—C8	1.5044 (15)	C21—H21A	0.9800
C8—C9	1.5204 (15)	C21—H21B	0.9800
C8—H8A	0.9900	C21—H21C	0.9800
			
C18—O2—C21	117.24 (9)	C1—C10—C11	119.10 (9)
C1—N1—C13	124.74 (9)	C1—C10—C9	118.18 (10)
C1—N1—H1	121.2 (10)	C11—C10—C9	122.72 (9)
C13—N1—H1	114.0 (9)	C12—C11—C10	118.93 (9)
N1—C1—C10	120.00 (10)	C12—C11—C15	120.07 (10)
N1—C1—C2	118.25 (9)	C10—C11—C15	120.99 (9)
C10—C1—C2	121.74 (9)	C11—C12—C14	122.63 (10)
C3—C2—C7	119.70 (10)	C11—C12—C13	121.90 (10)
C3—C2—C1	122.49 (10)	C14—C12—C13	115.35 (9)
C7—C2—C1	117.81 (10)	O1—C13—N1	120.54 (10)
C4—C3—C2	120.28 (10)	O1—C13—C12	124.44 (10)
C4—C3—H3	119.9	N1—C13—C12	115.01 (9)
C2—C3—H3	119.9	N2—C14—C12	177.04 (11)
C3—C4—C5	119.97 (11)	C16—C15—C20	118.30 (10)
C3—C4—H4	120.0	C16—C15—C11	120.52 (10)
C5—C4—H4	120.0	C20—C15—C11	121.18 (10)
C6—C5—C4	120.10 (11)	C17—C16—C15	121.76 (10)
C6—C5—H5	119.9	C17—C16—H16	119.1
C4—C5—H5	119.9	C15—C16—H16	119.1
C5—C6—C7	120.81 (10)	C16—C17—C18	118.99 (10)
C5—C6—H6	119.6	C16—C17—H17	120.5
C7—C6—H6	119.6	C18—C17—H17	120.5
C6—C7—C2	119.13 (10)	O2—C18—C17	124.26 (10)
C6—C7—C8	122.27 (10)	O2—C18—C19	115.50 (10)
C2—C7—C8	118.55 (10)	C17—C18—C19	120.24 (10)
C7—C8—C9	111.49 (9)	C20—C19—C18	119.96 (10)
C7—C8—H8A	109.3	C20—C19—H19	120.0
C9—C8—H8A	109.3	C18—C19—H19	120.0
C7—C8—H8B	109.3	C19—C20—C15	120.75 (10)
C9—C8—H8B	109.3	C19—C20—H20	119.6
H8A—C8—H8B	108.0	C15—C20—H20	119.6
C10—C9—C8	110.71 (9)	O2—C21—H21A	109.5
C10—C9—H9A	109.5	O2—C21—H21B	109.5
C8—C9—H9A	109.5	H21A—C21—H21B	109.5
C10—C9—H9B	109.5	O2—C21—H21C	109.5
C8—C9—H9B	109.5	H21A—C21—H21C	109.5
H9A—C9—H9B	108.1	H21B—C21—H21C	109.5
			
C13—N1—C1—C10	3.20 (16)	C1—C10—C11—C15	175.99 (9)
C13—N1—C1—C2	−176.95 (9)	C9—C10—C11—C15	−4.29 (16)
N1—C1—C2—C3	−18.44 (15)	C10—C11—C12—C14	175.55 (9)
C10—C1—C2—C3	161.41 (10)	C15—C11—C12—C14	−3.61 (16)
N1—C1—C2—C7	161.30 (10)	C10—C11—C12—C13	−0.36 (16)
C10—C1—C2—C7	−18.86 (15)	C15—C11—C12—C13	−179.53 (9)
C7—C2—C3—C4	0.04 (16)	C1—N1—C13—O1	174.67 (10)
C1—C2—C3—C4	179.77 (10)	C1—N1—C13—C12	−6.45 (15)
C2—C3—C4—C5	0.84 (17)	C11—C12—C13—O1	−176.24 (10)
C3—C4—C5—C6	−0.96 (18)	C14—C12—C13—O1	7.56 (16)
C4—C5—C6—C7	0.19 (18)	C11—C12—C13—N1	4.93 (15)
C5—C6—C7—C2	0.69 (17)	C14—C12—C13—N1	−171.27 (9)
C5—C6—C7—C8	−176.55 (11)	C12—C11—C15—C16	128.40 (11)
C3—C2—C7—C6	−0.80 (16)	C10—C11—C15—C16	−50.74 (14)
C1—C2—C7—C6	179.46 (10)	C12—C11—C15—C20	−51.08 (14)
C3—C2—C7—C8	176.55 (10)	C10—C11—C15—C20	129.78 (11)
C1—C2—C7—C8	−3.20 (15)	C20—C15—C16—C17	0.34 (16)
C6—C7—C8—C9	−144.07 (11)	C11—C15—C16—C17	−179.16 (10)
C2—C7—C8—C9	38.67 (14)	C15—C16—C17—C18	−0.89 (16)
C7—C8—C9—C10	−52.36 (12)	C21—O2—C18—C17	−4.32 (17)
N1—C1—C10—C11	1.94 (15)	C21—O2—C18—C19	175.79 (10)
C2—C1—C10—C11	−177.91 (9)	C16—C17—C18—O2	−179.15 (10)
N1—C1—C10—C9	−177.80 (9)	C16—C17—C18—C19	0.73 (16)
C2—C1—C10—C9	2.36 (15)	O2—C18—C19—C20	179.86 (10)
C8—C9—C10—C1	33.28 (13)	C17—C18—C19—C20	−0.04 (17)
C8—C9—C10—C11	−146.44 (10)	C18—C19—C20—C15	−0.53 (17)
C1—C10—C11—C12	−3.17 (15)	C16—C15—C20—C19	0.38 (16)
C9—C10—C11—C12	176.55 (10)	C11—C15—C20—C19	179.87 (10)

### Hydrogen-bond geometry (Å, °)

**Table:**

*D*—H···*A*	*D*—H	H···*A*	*D*···*A*	*D*—H···*A*
N1—H1···O1^i^	0.90 (2)	1.94 (2)	2.823 (1)	166 (1)

Symmetry codes: (i) −*x*+1, −*y*+1, −*z*+1.
